# Barriers to international physician recruitment in Nova Scotia, Canada

**DOI:** 10.1177/08404704231165727

**Published:** 2023-03-31

**Authors:** Karuna Manchanda, Matthew Murphy

**Affiliations:** 1 3688 Trillium Health Partners, Mississauga, Ontario, Canada.; 2 432234 Nova Scotia Health Authority, Antigonish, Nova Scotia, Canada.

## Abstract

A healthcare staffing crisis has been brewing in Canada since 1993. Recently worsened by the COVID-19 pandemic and increasing immigration, it has severely impacted rural and remote areas of the country like the province of Nova Scotia. Researchers have considered international physician recruitment as a long-term solution, but it comes with its own challenges. In addition to an extensive literature search, qualitative interviews were conducted with various representatives from the Nova Scotia health ecosystem as part of this article. Identifying challenges to international physician recruitment from different perspectives, recommendations include bringing legislative and/or policy changes to increase candidate seats and developing new pathways to bring international medical graduates to Nova Scotia from other countries. The article includes interview responses from official authorities involved in physician recruitment, author recommendations to remove barriers to international physician recruitment, and recruitment and retention initiatives currently being implemented in the province.

## Background

Dating back to as far as 1993, Canada’s population has been facing a shortage of physicians, especially in the rural and remote areas.^1^ This shortage of physicians and other healthcare workers has been gradually increasing and has currently reached an all-time high.^2^ Nova Scotia (NS), being predominantly rural, has borne the brunt of this situation in 2021 more than other provinces with more than 2,100 healthcare vacancies reported.^3^ With the increasing vacancies, in 2016, 36,000 people had signed up in the province’s “Need a Family Practice” registry.^4^ In 2021, the census recorded 969,383 people living in the province of Nova Scotia.^17^ The last reported number signed up on the registry was 125,000 (people waiting for a family doctor in Nova Scotia) in December 2022 which can only translate to a much higher number including those who are not signed up.

Several articles have identified issues regarding the physician shortage in Canada as well as offered solutions; however, not much has come into effect in terms of visible improvements. This article focuses on international physician recruitment, one of the previously identified solutions.^5^

From immigration challenges to licensure delays, there are many challenges at each step of the physician recruitment and retention process in Nova Scotia. Various authorities in Nova Scotia work collaboratively to facilitate recruitment. These authorities are the Department of Health & Wellness (DHW), Nova Scotia Health Authority (NSH), The College of Physicians and Surgeons of Nova Scotia (CPSNS), Doctors Nova Scotia (DNS), Immigrant Services Association of Nova Scotia (ISANS), and a newly created Office of Healthcare Professionals Recruitment (OHPR). While the DHW is responsible for funding most initiatives as well as physician compensation, the NSH’s Medical Affairs portfolio holds the responsibility of attracting physicians to NS and fast-tracking their applications as well as their settlement into the recruited communities. Licensure, practice assessments, and final decisions regarding a physician’s qualifications are carried out by the CPSNS. ISANS is responsible for granting visas or work permits to eligible physicians. DNS is the entity that acts as a union front for the physicians. Due to the involvement and need for collaboration from a number of entities, it becomes challenging to coordinate and proceed in a timely manner. The new Office of Healthcare Professionals Recruitment was created to help mediate and coordinate across these various entities.^6^

## Methodology

For the purposes of this study, an extensive literature search was conducted via Google Scholar, PubMed, and PubMed Central databases. Nine studies were included in this literature review after conducting a directed content analysis. The inclusion criteria consisted of recruitment and retention of physicians in Nova Scotia, Canada. Keywords used were recruitment, barriers, and physicians. In addition, a total of five semi-structured interviews were conducted with the following professionals: CEO/Registrar of The College of Physicians and Surgeons of Nova Scotia, personnel from the Office of Healthcare Professionals Recruitment, President of Doctors Nova Scotia, an experienced physician in NS, and an International Medical Graduate (IMG) post-graduate at Dalhousie University. Informed consent was obtained before the interviews, and a draft of this article was reviewed by interviewees before manuscript submission. The interviews were conducted with a qualitative approach via on-line platforms, and the duration was approximately one hour each. The interviewees were experienced healthcare professionals of both genders. The interviews included questions pertaining to the background of the individual, role of their organization in physician recruitment, their perspective on the challenges to international physician recruitment in NS, possible solutions, and their knowledge of any policies/processes that are currently being implemented/changed to support recruitment and retention.

## Literature review

Via immigration, Canada’s population has been increasing over the years, yet the physician-to-population ratio has not improved. In the period between 2003 and 2013, the percentage of population (aged 15 and older) facing difficulty accessing first-contact healthcare services varied from 16% in 2003 to 17% in 2013 in terms of health information or advice.^7^ The fact that this percentage hardly improved in 10 years is evidence that there is a physician shortage in the country.

Nova Scotia, being primarily rural, is facing a major shortage of family physicians and it is important to assess the different factors that play a role in physician recruitment and retention in rural areas.^8^ There are 30 years of literature studies supporting the fact that rural Canada faces physician shortage and there are several barriers to international physician recruitment contributing to this shortage.^2,5,8-12^

When it comes to international physician recruitment in Nova Scotia, there are several factors that act as barriers. International Medical Graduates (IMGs) can only obtain observer positions in NS before they can have a licence to practice medicine.^13^ However, without hands-on experience, it is almost impossible for IMGs to attain full licensure.^10^ Furthermore, IMGs also must apply for a work permit, social insurance number, driver’s licence, etc. on top of the regular paperwork required by the CPSNS.^8^

The physician recruitment department in Nova Scotia is currently working with other previously mentioned authorities to understand these factors and find alternative routes to support IMG recruitment.^6^ The interviews conducted in the study by Macneill et al. suggested there was a “disconnect” between participants from different authorities.^8^ One of the solutions to this issue is to create a more effective recruitment office that clearly defines roles and responsibilities,^8^ which is now evident in the actions taken by the newly elected government in 2021 as they created the new Office of Healthcare Professionals Recruitment.^6^

With respect to international recruitment to rural areas, an important factor to address is the ethical concern relating to the practice.^5^ Since international physicians are mostly being recruited to rural settings, there are existing concerns about whether the physicians are being recruited to good working conditions in developed nations.^5^ Rural recruitment comes with its own pitfalls including standard of life or flexibility of work hours and vacations.^5^ However, IMGs choose to leave their home countries for a better life, wages, and security in Canada and that choice is constituted within their personal autonomy and basic human rights.^5^

## Results

### Existing recruitment efforts

CPSNS was established to serve the purpose of protecting the public and holding doctors accountable, and it believes that an increase in recruitment cannot be brought about by lowering the standards for acceptance to practice in the country. CPSNS is looking into recruiting Canadian students graduating from medical schools abroad, assessing ways to fast-track their candidature to licensure in NS. The College also corrected the popular misconception by clarifying that, “Source verification of documents does not cause delays in international recruitment; the rate-limiting step is immigration.”

“The Office was established to act as an umbrella of support for the existing authorities involved in recruitment activities”, said the personnel. While the OHPR does not recruit directly, it does determine the needs of the communities in terms of physician recruitment. There is no global recruitment target for the province (in terms of a target number of physicians to be recruited monthly or annually); however, they do have local objectives as per the needs of the communities.

The role of DNS is to provide support to physicians after they have been recruited to work in NS. From enrolling physicians into mentorship programs to supporting current medical students and post-graduate trainees at Dalhousie University, DNS works to allow current and future physicians access the benefits they are entitled to.

### Legislation and policy

CPSNS believes that legislative changes are not going to help solve the health staffing shortage in Nova Scotia; however, policy changes may help. The Office confirmed that policy development is ongoing from their end, but no legislation will be changed. The OHPR has several partnerships in progress with ISANS, DHW, Chamber of Commerce, NSH, CPSNS, Dalhousie University, and community-level partnerships.

### Current challenges

It is a common understanding that international recruitment challenges vastly lie in cultural barriers that need to be broken down. “Cultural integration needs to be achieved in the actual practice of medicine. It leads to a sense of belonging. However, there needs to be a degree of community investment,” said the CEO of CPSNS. Physician mentorship programs have been launched by CPSNS to help settle new physicians in their communities. Some challenges with physician mentorship programs include recruiting physician mentors from the system to mentor incoming physicians at the cost of patient care. Additionally, CPSNS struck an agreement with ISANS four years ago that allowed IMGs to go through an expedited process to obtain work permits in NS. The agreement drastically improved immigration challenges faced by IMGs in the province.

The physician interviewees believed that some major challenges include the lack of succession planning in the NS healthcare ecosystem, the gap in source verification, and recognition of a limited number of jurisdictions. DNS believes challenges with expanding the Practice Ready Assessment Program (PRAP) in NS include (i) the scarcity of supervisors and assessors participating in the program, (ii) the limited number of facilities that can be used as assessment sites for the program, and (iii) the limited supply of appropriately qualified IMGs to be recruited through the PRAP. The President of DNS believes the biggest challenge to IMG recruitment is integrating them into rural communities. DNS works through this challenge by enrolling physicians into mentorship programs. The President of DNS mentioned that physicians often leave or relocate after they are recruited, and DNS is working to understand the reasons why they leave so they can work with communities to improve future physician retention.

### Current and future initiatives

The personnel from the OHPR mentioned the NS Physician Incentive Program being implemented in March 2022 under two streams—Family Physician and Royal College Specialist streams (with an increase in incentives being offered to physicians in deprived communities to improve retention). Interim evaluations will be held for this policy in the next five years. The OHPR also confirmed that the remuneration model policy for physicians is to be expanded to grant the Alternative Payment Plan (APP) to all physicians who request for it. Some policy changes to be implemented by the OHPR in the future include removing the “one in one out” rule for physicians in the province abolishing systemic bureaucracy. Payment models will be upgraded to a hybrid version of fee-for-service and contract models, becoming more of a blended capitation model. Moreover, a new MD Recruiter Program will be launched—hiring physicians from the system to find out the current situation and barriers to recruitment from recent recruits.

From DNS, the Physician Mentorship Program – Compass and Study Support programs have been implemented (for new physician mentorship, guidance, and support).^14^ “The priority group that we have started to prioritize first is the international medical graduates and the PRAP graduates to figure out exactly what supports are going to be most helpful,” said DNS’s President. DNS has conducted a research study titled, “The Future of Family Medicine: Nova Scotia family physicians’ vision and priorities.”^15^ From the responses received, it is evident that physicians want the following—develop e-health infrastructure, increase access to blended capitation, collaborative teams, and increased family physician engagement in system-level decision-making.^15^ Under future initiatives, a Health Leadership Council is to be created with representatives from the Ministry of Health, Nova Scotia Health, Office of Healthcare Professionals Recruitment, and other authorities. “The sole focus of this council will be on bringing team-based care into practical existence in the province,” said DNS’s President.

## Recommendations

In terms of existing and upcoming policies, it is apparent that the authorities are moving in the right direction. However, as mentioned by the physicians currently in the system, there are other aspects beyond financial incentives that are being overlooked by the authorities. These include expanding the seats in the Practice Ready Assessment Program to include other specialities, increasing candidate seats matching through the Canadian Resident Matching Service (CaRMS), establishing agreements with countries whose medical education systems resemble the Canadian system for faster recruitment, posting advisors at international medical schools to bring Canadian medical graduates to NS, identifying curriculum similarities internationally to develop new pathways for medical graduates to transition into the Canadian healthcare system, introducing alternative fellowship routes for international physicians to enter the healthcare system in the province, and accommodating spousal employment for physicians willing to relocate to rural communities. Progress is observed in NS as it becomes the first province in Canada to recognize credentials of US board-certified doctors without exams.^16^ This change, been brought on by CPSNS recently, will significantly remove barriers for international physicians coming to NS from the United States. Policy directives that may help in the future can include ones focusing on increasing spousal employment and support for recruited physicians, removing cultural barriers from rural settings, maintaining and propagating the physician mentorship programs, and abolishing the bureaucracy around the “one in one out” rule.

## Discussion

It is clear from the literature review as well as the interview responses that there is a major healthcare staffing crisis in NS. The healthcare system in the province is burdened and understaffed, and while there are many possible solutions to the problem, one of them remains increasing international recruitment. Deriving from the information collected and reported above, it is evident that the major authorities involved with international physician recruitment in NS are aware of all the barriers that exist for the same. The challenges are perceived differently by all, and with the College believing no major legislative changes are required, future course-altering solutions may have to come from the OHPR, NHS, or DHW. International physicians face many challenges that are often not faced by Canadian graduates; and to help them overcome these challenges, the authorities are required to develop and implement new initiatives.

There are two limitations to the information collected in this article. Firstly, there was a lack of documentation provided by the authorities to support their statements and actions (current and future). When inquired for policy documents, there was hesitation by other authorities, but a few official documents were shared for the purposes of this article by DNS. The College and the OHPR showed restraint in terms of sharing official policies, legislative documents, implementation proofs, however, the OHPR shared documents of their newest initiatives. Secondly, singular officials from authorities like DNS, CPSNS, and the OHPR were interviewed which may have introduced some biased views in their comments to show support towards their respective organizations.

## Conclusion

Overall, physician recruitment and retention comes with many complex challenges that need to be solved with a holistic view of the situation. With several authorities working together to recruit and support physicians in the province, there needs to be a certain degree of collaboration and communication for effective elimination of barriers. Areas for future research may include evaluation of the impact of current actions taken by the new Office of Healthcare Professionals Recruitment, barriers to increasing training slots or expanding the PRAP in the province and comparing Nova Scotia to other Canadian provinces and their physician recruitment programs. With visibly increased efforts being directed to improving international physician recruitment and retention in Nova Scotia, it is vital to develop evaluation processes to monitor the progress and impact of these efforts.
